# A Cost-Effective Inertial Measurement System for Tracking Movement and Triggering Kinesthetic Feedback in Lower-Limb Prosthesis Users

**DOI:** 10.3390/s21051844

**Published:** 2021-03-06

**Authors:** McNiel-Inyani Keri, Ahmed W. Shehata, Paul D. Marasco, Jacqueline S. Hebert, Albert H. Vette

**Affiliations:** 1 Donadeo Innovation Centre for Engineering, Department of Mechanical Engineering, University of Alberta, 9211 116 Street NW, Edmonton, AB T6G 1H9, Canada; mcniel@ualberta.ca; 2 Division of Physical Medicine and Rehabilitation, Faculty of Medicine and Dentistry, University of Alberta, 5005 Katz Group Centre, Edmonton, AB T6G 2E1, Canada; shehata@ualberta.ca (A.W.S.); jhebert@ualberta.ca (J.S.H.); 3 Laboratory for Bionic Integration, Department of Biomedical Engineering, Lerner Research Institute, Cleveland Clinic, 9500 Euclid Avenue, ND20, Cleveland, OH 44195, USA; marascp2@ccf.org; 4 Advanced Platform Technology Center, Louis Stokes Cleveland Department of Veterans Affairs Medical Center, 10701 East Boulevard 151 W/APT, Cleveland, OH 44106, USA; 5 Department of Biomedical Engineering, 1098 Research Transition Facility, University of Alberta, Edmonton, AB T6G 2V2, Canada; 6 Glenrose Rehabilitation Hospital, Alberta Health Services, 10230 111 Avenue NW, Edmonton, AB T5G 0B7, Canada

**Keywords:** device development, kinematic feedback, kinesthetic feedback, kinesthetic illusion, lower-limb prostheses, sensory feedback, wearable sensor

## Abstract

Advances in lower-limb prosthetic technologies have facilitated the restoration of ambulation; however, users of such technologies still experience reduced balance control, also due to the absence of proprioceptive feedback. Recent efforts have demonstrated the ability to restore kinesthetic feedback in upper-limb prosthesis applications; however, technical solutions to trigger the required muscle vibration and provide automated feedback have not been explored for lower-limb prostheses. The study’s first objective was therefore to develop a feedback system capable of tracking lower-limb movement and automatically triggering a muscle vibrator to induce the kinesthetic illusion. The second objective was to investigate the developed system’s ability to provide kinesthetic feedback in a case participant. A low-cost, wireless feedback system, incorporating two inertial measurement units to trigger a muscle vibrator, was developed and tested in an individual with limb loss above the knee. Our system had a maximum communication delay of 50 ms and showed good tracking of Gaussian and sinusoidal movement profiles for velocities below 180 degrees per second (error < 8 degrees), mimicking stepping and walking, respectively. We demonstrated in the case participant that the developed feedback system can successfully elicit the kinesthetic illusion. Our work contributes to the integration of sensory feedback in lower-limb prostheses, to increase their use and functionality.

## 1. Introduction

Limb loss affects many individuals across the world, with the majority of affected individuals experiencing it in the lower extremity [1,2], thereby affecting mobility and gait. Since the degree of mobility has been shown to have a positive correlation with the overall quality of life of individuals with lower limb loss [3], various prosthetic technologies have been developed to restore ambulation [4]. Nonetheless, lower-limb prosthesis users still suffer from decreased balance during walking, which can contribute to a higher incidence of falling, abnormal gait, and a decreased quality of life [5]. One cause for this might be the loss of proprioceptive feedback such as kinesthesia (sense of limb and joint movement), which, when restored following limb loss, has been shown to result in greater joint placement control and reflexive behavior during stair walking [6]. As a consequence, prostheses capable of providing users with a relative sense of the prosthetic limb’s movement could improve mobility, reduce fall risk, and contribute to an improved quality of life for affected individuals.

The kinesthetic illusion (KI), a phenomenon whereby mechanical vibration administered to the musculotendinous region of a limb may elicit a perception of limb movement, holds promise as a means of providing sensory feedback on movement to prosthesis users [7]. This perceptual phenomenon can be triggered when vibrating a limb’s muscle belly or tendon at a frequency between 70 and 115 Hz to simulate muscle contraction [8,9,10]. For example, vibration introduced to the lower limbs of non-disabled individuals has been shown to result in vibration-induced falling and a perception of forward progression during stationary marching [11,12,13]. Thus far, the KI has been demonstrated in non-disabled individuals [8,11], upper-limb prosthesis users [14], and, more recently, lower-limb prosthesis users [9]. While the KI is commonly administered manually using a hand-held actuator capable of eliciting movement perceptions [7,10,15], its functional use requires automated feedback control, based on the sensed movement of a user’s prosthesis. A challenge with achieving this automated kinematic feedback, however, is the lack of adequate instrumentation that can measure and relay kinematic information of the lower-limb prosthesis to its user, and this in an accurate, reliable, timely, and cost-effective fashion.

Inertial measurement units (IMUs), technology frequently used for tracking human and robotic position and orientation [16,17,18], can be found within a minor cohort of advanced commercial prostheses. Although such microprocessor-controlled lower-limb prostheses rely on IMUs to monitor the prostheses’ movement and facilitate their optimal performance (e.g., by adapting to varying terrains [4]), the majority of lower-limb prostheses do not [19,20]. Additionally, acquiring movement data wirelessly from advanced prostheses is difficult without compromising their integrity and making physical modifications to the system [21]. Thus, retrofitting prosthetic devices with easy-to-attach, low-cost, wireless IMUs may aid researchers in acquiring prosthesis movement data, for use in closed-loop feedback systems utilizing the KI. However, when retrofitting current prosthetic technologies with commercially available tracking systems, e.g., the Delsys Trigno, the high cost of such tracking systems and the high processing power required present as additional practical challenges.

The lack of instrumentation of most prostheses, the inaccessibility of data from the subset of more advanced prosthetic devices, and the high cost of commercial tracking systems prevent researchers from developing a functional method of integrating sensory feedback systems while monitoring movement of the prosthesis. In this light, the first objective of this study was to develop a low-cost wireless IMU-based system (WIbS), capable of driving a vibratory actuator in a feedback control scheme. The second objective of this study was to investigate the feasibility of using the developed feedback system to trigger the KI, in real-time, in a case study with an above-knee lower-limb prosthesis user.

## 2. Materials and Methods

### 2.1. Device Development

**Figure 1:**
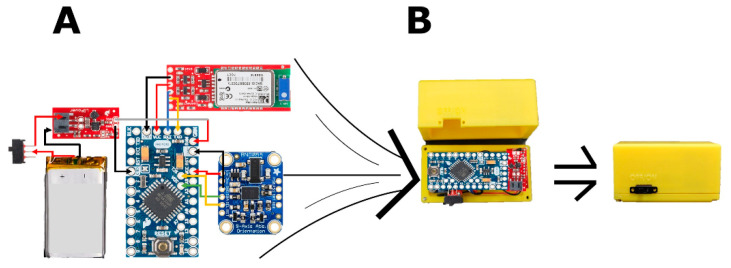
(**A**) The wireless inertial measurement unit-based system (WIbS) consisted of two of the following: an Arduino Pro Mini microcontroller (center), an inertial measurement unit (bottom right), a Bluetooth radio (top right), a lithium-ion battery, and a boost converter (left of the microcontroller). (**B**) An enclosure was manufactured for each sensor module to manage wires, improve ergonomics, and fixate the inertial measurement unit.

**Hardware and enclosure:** The developed, low-cost WIbS is comprised of an Arduino Pro Mini microcontroller (Arduino, Somerville, MA, USA), an RN42 Bluetooth module (Microchip Technology, Chandler, AZ, USA) capable of delivering up to three megabits per second of data at distances of up to 20 m, two IMU modules with three rotational degrees of freedom (BOSCH, Gerlingen, Germany), and a lithium-ion battery with a boost converter offering up to four hours of battery life (SparkFun, Niwot, CO, USA) (Figure 1A). We specifically chose IMUs with three rotational degrees of freedom as this choice will allow application to prosthetic knee or ankle joints in the future that facilitate motion of more than a single degree of freedom (e.g., ankle flexion/extension and inversion/eversion [22,23]). The cost of the described hardware is approximately US $100. Due to the modularity of the system, other electronic components with similar or superior specifications could be easily substituted into the system.

To manage wires, improve ergonomics, and most importantly fixate each of the two IMU modules to the prosthesis for accurate measurements, two enclosures (70 mm × 35 mm × 35 mm each) were designed using SolidWorks 2016 (SOLIDWORKS, Waltham, MA, USA) (Figure 1B). They were manufactured via three-dimensional printing using fused deposition rapid prototyping, polylactic acid filaments (MakerBot, NY, USA), and the MakerBot Replicator (MakerBot, Brooklyn, NY, USA).

**Data transmission:** The microcontroller logs four unit quaternions at a sampling rate of 100 Hz from each IMU and transmits these wirelessly to a mobile monitoring station at a baud rate of 9600 bits per second. The unit quaternions are contained in a data structure of 14 bytes: two leading bytes (flags), eight bytes storing the four unit quaternions, and the calibration status of the given IMU’s gyroscope (1 byte), accelerator (1 byte), magnetometer (1 byte), and overall system (1 byte).

**Prosthetic joint angle calculation:** To determine the three-dimensional angles of a prosthetic joint, the two IMU modules of the WIbS must be placed on rigid segments of the prosthesis, proximally and distally of the joint of interest. The time-varying orientation of each IMU module is computed from rotation matrices obtained via the device’s unit quaternions [24]. Following placement, the WIbS is calibrated to virtually align the coordinate frames of both IMUs with relevant rotation axes of the prosthesis. To do so, a constant calibration matrix is obtained for each IMU that captures its orientation relative to the underlying segment when the segment’s orientation in the global coordinate system is known [25]. The IMUs’ rotation matrices during movement trials, obtained according to [24], are then calibrated via respective calibration matrices. The result is then used to compute, via Equation (1), a rotation matrix yielding the angular kinematics between the two prosthetic limb segments:

*R_joint angle_* = *R^T^_proximal limb segment_* × *R_distal limb segment_*. (1)

Finally, Cardan angles yielding the three-dimensional angles of the prosthetic joint are calculated using the Z-X-Y moving axes sequence, as recommended by the International Society of Biomechanics [26]. Since most lower-limb prosthetic joints are single-axis joints [4], the risk of encountering gimbal lock is negligible [27].

**Software:** We used the C# programming language to design a graphical user interface (GUI) that logs and visualizes data received from the WIbS. Cardan angles representing the monitored joint angles (e.g., of the knee) as well as the calibration status of each IMU are shown in the GUI. Quaternions, calibration status, recording time of the WIbS, and the three-dimensional joint angles (e.g., of the knee) acquired via the WIbS are logged at a sampling rate of 33 Hz.

### 2.2. Measurement Validation

**Experimental approach and procedures:** We quantified the accuracy of the WIbS relative to the outputs of an optoelectronic motion capture system (OptiTrack, Corvallis, OR, USA) and a commercial IMU (cIMU) system (Delsys, Natick, MA, USA). These two systems acquired motion data at sampling frequencies of 120 Hz and 33 Hz, respectively. Considering again that most lower-limb prosthetic joints are single-axis joints [4], we employed a robotic arm [28] in a bench test due to the fact that it can facilitate repeatable and isolated single-axis WIbS motion. We attached each WIbS IMU module to a rigid motion capture plate that also held four motion capture markers and a cIMU system. We placed one of the two WIbS IMU modules on a stationary segment of the robotic arm, and the other one on a segment that could move about the three IMU axes.

We used Gaussian and sinusoidal movement velocity profiles (three trials per axis and per velocity) to assess the accuracy and repeatability of the WIbS. Accordingly, a total of 54 trials were performed (3 trials × 3 axes × 3 velocities × 2 profiles). We chose the Gaussian and sinusoidal movement profiles since previous work has shown that they model single steps and are representative of the cyclic nature of walking, respectively [29]. The single-axis joint tracking system was tested three times for the maximum velocities of 60, 120 and 180 degrees/s, i.e., velocities and associated accelerations that are in line with the gait speed and cadence for prosthesis users during walking [30,31,32,33]. For the Gaussian movement profile, the velocity features a double peak, with the first being positive and the second negative; the acceleration features three peaks, with the first and last being smaller and positive, and the second being larger and negative. For the sinusoidal movement profile, the velocity and acceleration will again follow sinusoidal profiles. For each of the tested velocities per axis, the average root-mean-square error (RMSE) was computed following previous protocols [34,35], but with slight adjustments for gait as shown in Table 1.

**Data processing and analysis:** Experimental data were processed in MATLAB 2016b (MathWorks, Natick, MA, USA). Using the calibration approach described in Section 2.1 as well as the WIbS data, the motion capture data, and the cIMU data, we obtained a time-varying rotation matrix that captures the instantaneous orientation of each robotic segment for each motion tracking system. We then computed joint angles of the robot, represented by Cardan angles, from the time-varying rotation matrix for each system.

We used the RMSE to determine the accuracy of the WIbS. To ensure a valid comparison, data from the WIbS (33 Hz) and the cIMU (33 Hz) were resampled to align their sampling rates with that of the OptiTrack motion capture system (120 Hz). Subsequently we used a second order, low-pass Butterworth filter with a cut-off frequency of 4 Hz to eliminate any high-frequency noise. The resultant signals were cross-correlated to align the signals temporally. Finally, RMSE values were calculated between the WIbS angles and the motion capture or cIMU angles. For both movement profiles and each velocity, RMSE values for single-axis movements were averaged across trials.

### 2.3. System Integration

**Triggering system using threshold-based controller:** A kinesthetic feedback system, utilizing the WIbS and a simple threshold-based controller, was developed to trigger a vibratory actuator (states: on or off) that has been shown to be capable of inducing the KI [9]. Using the same hardware and technique as those used to transmit unit quaternions from the WIbS to the GUI (see Section 2.1), the developed system processes movement data and wirelessly controls the vibratory actuator. More specifically, the threshold-based controller infers movement by comparing the angular joint velocity (determined using the WIbS) to a predetermined threshold of 1.65 degrees per second (0.05 degrees × 33 Hz). The threshold value was chosen to test the WIbS’s sensitivity in a controlled environment. Practical use of the movement sensor might permit a larger threshold, e.g., based on the angular velocity of both intact and prosthetic knee joints [30,33,36], to prevent false movement detection. False movement detection due to high-frequency noise is eliminated by a fifth order moving average filter with a cut-off frequency of 4 Hz.

### 2.4. Case Study: Above-Knee Prosthesis User

**Participant and experimental setup:** Experiments were conducted to demonstrate the developed system’s ability to elicit movement percepts in a participant with lower limb loss that has previously experienced the KI. A 19-year-old, male individual who had a transfemoral amputation performed 18 months prior (due to osteosarcoma) participated in this study. He had no current or previous phantom limb pain, used a passive hydraulic knee, and experienced no current or previous neurological or muscular health conditions, other than the limb loss. The participant provided written informed consent prior to participating in the study, which was approved by the Health Research Ethics Board at the University of Alberta (Pro00063695).

**Figure 2:**
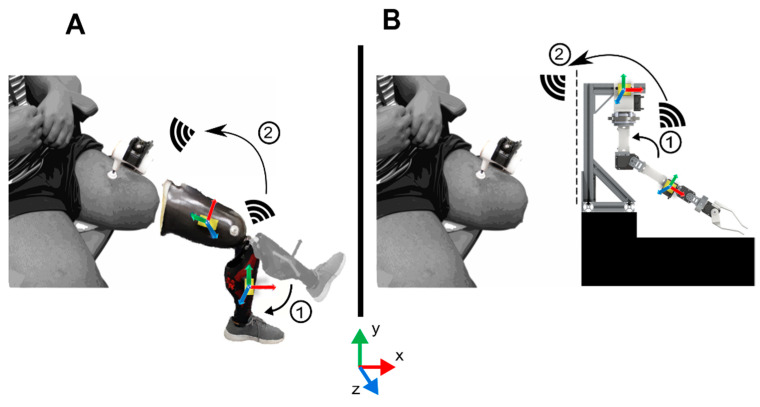
(**A**) The participant was shown his unattached prosthesis with the inertial measurement unit (IMU) modules attached proximally and distally of the knee joint. For each segment of the prosthesis, the orientation of the IMU module was calibrated as described in Section 2.1. The participant was informed that movement of the prosthesis, as detected by the wireless modules, was responsible for triggering the vibratory actuator. (**B**) The participant was unaware, however, that the robotic arm was used instead to provide motion measurements, triggering the vibration motor. The calibrated local coordinate frames and the global coordinate frame are shown within and below the figure, respectively.

The experimental setup consisted of two WIbS IMU modules attached proximally and distally of a single joint of the robotic arm from Section 2.2, which communicated wirelessly with a vibration motor (VB200, Vibrasens, Techno Concept, Manosque, France) featuring a flat-faced probe tip (2.7 cm diameter) clamped on the participant’s thigh (Figure 2). Since the case study’s goal was to demonstrate the developed system’s ability to induce the KI in a controlled environment, we strived to minimize external influences such as motion artifacts. Therefore, the automated robotic arm was used for consistency and repeatability in limb movement as well as activation of the vibration motor across trials.

**Experimental procedures and data analysis:** The experimental protocol was divided into two parts: percept mapping and illusion quantification. On the one hand, percept mapping was used to identify a site on the participant’s residual limb that elicited strong and consistent movement percepts with vibration. On the other hand, illusion quantification was used to characterize the nature, velocity, and duration of the movement illusion.

For the *percept mapping*, we followed our previously published protocol [7,9] that required the participant to wear a blindfold and noise-cancelling headphones playing Brownian noise to occlude visual and auditory cues, respectively. The vibration device was pressed into different locations of the muscle bellies of the vastus lateralis, rectus femoris, and vastus medialis muscles on the participant’s residual limb. For each location, we asked the participant to indicate any sensation beyond simple vibration. Sites where the participant perceived an illusory movement sensation were marked. We also asked the participant to rate the realism of each illusory movement on a psychophysical 5-point Likert scale [37] from 1 (a weak movement illusion) to 5 (a strong movement illusion).

Once the location with the strongest and most consistent movement percept during vibration was identified (with the prosthesis detached), it was used in the *illusion quantification*. Since the KI illusion is a psychophysical phenomenon, guiding participants to visually link the movement of their prosthesis to the movement percept has the potential to enhance the KI [38]. Therefore, an initial demonstration of the triggering system was conducted that had the WIbS fixated to the detached prosthesis and tracking its movement. The demonstration aimed to convince the participant that knee joint movement associated with his prosthesis, as detected by the WIbS, was responsible for driving the vibratory actuator. However, while the participant’s vision was occluded, the IMU modules were transferred onto the concealed robotic arm, for the reasons stated above. A set of empty enclosures, identical to those used by the WIbS, were then attached to the same locations on the detached prosthesis to convince the participant, whenever vision was unconcealed, that the movement of his prosthesis was still responsible for activating the vibration motor.

20 trials with the developed feedback system were executed during illusion quantification. Prior to executing those trials, the participant was asked to wear a blindfold and noise-cancelling headphones playing Brownian noise to occlude visual and auditory cues, respectively. For each trial, the robotic arm followed half a Gaussian profile (0 to 90 degrees) for a duration of 15 s, and the clamped vibration motor was triggered by the WIbS tracking the single-axis movement of the robot. More specifically, the onset and termination of the robotic arm’s movement were used to activate or deactivate the vibration motor, respectively (see Section 2.3). Upon activation, the vibration motor provided stimulation at parameters previously determined to be optimal for elicitation of the KI–a frequency of 90 Hz and an amplitude of 1 mm peak-to-peak [7]. While the movement profile did not affect the actual vibration, a duration of 15 s was chosen since previous studies have found that, for the vibration parameters used in this work, the illusory movement sensation may sometimes last between 10 and 20 s [7,9,15]. For each trial, the participant was instructed to use his intact limb to match what he felt in terms of the nature, velocity, and duration of the movement. To quantify the illusion, we used a motion capture system (OptiTrack, OR, USA), along with motion capture plates placed proximally and distally of the participant’s intact knee, as well as the 5-point Likert scale mentioned above [37]. Once motion capture data and the strength of illusion were recorded, Cardan angles representing the knee joint angles were computed using the tools and techniques outlined in Section 2.1 and Section 2.2. To demonstrate the developed feedback system’s ability to elicit movement percepts, the knee flexion trajectories from the participant’s intact limb (used to match the kinesthetic perception) were then averaged across those trials that successfully elicited the KI. We then plotted the average knee flexion over time, along with its variability across trials (±1 standard deviation band). No further analysis was performed with the rating obtained for the psychophysical 5-point Likert scale.

## 3. Results

### 3.1. Movement Sensor Validation

**Figure 3:**
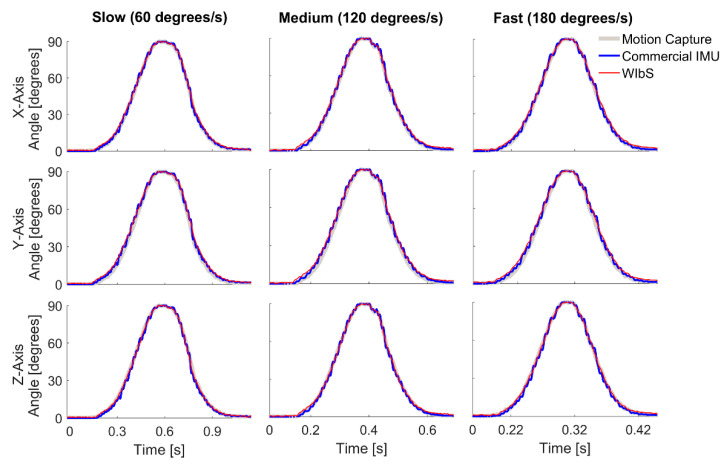
The Gaussian profile results for the individual axes of the wireless inertial measurement unit-based system (WIbS), red lines, when compared to those of the commercial inertial measurement unit system (cIMU), blue lines, and the motion capture system, gray lines. The rows show results for individual axes, whereas the columns represent the tested velocities.

**Figure 4:**
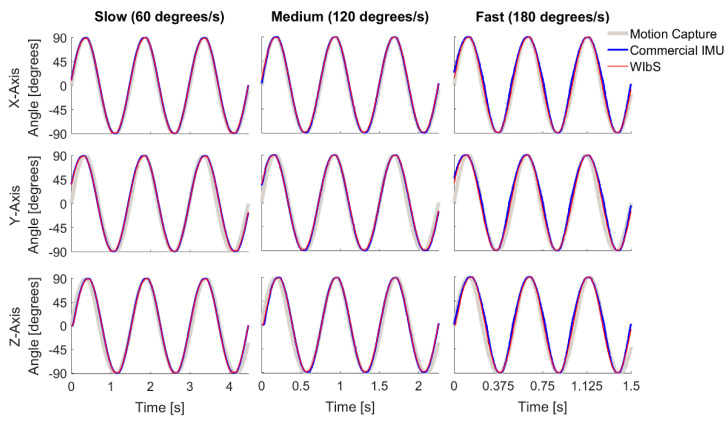
The sinusoidal profile results for the individual axes of the wireless inertial measurement unit-based system (WIbS), red lines, when compared to those of the commercial inertial measurement unit system (cIMU), blue lines, and the motion capture system, gray lines. The rows show results for individual axes, whereas the columns represent the tested velocities.

The performance of the WIbS is summarized in Figure 3 and Figure 4 and Table 2 and Table 3. In each plot, the red lines represent angles from the WIbS, blue lines angles from the cIMU system, and gray lines angles from the motion capture system. Rows represent results of a specific axis of rotation (X, Y, Z), and columns represent results for different velocities (60, 120, 180 degrees per second). For the Gaussian profile (Figure 3, Table 2), RMSE values were smaller than 1.0 and 0.5 degrees relative to the cIMU and motion capture measurements, respectively. The RMSE values for the angles of both stationary axes were smaller than 0.4 and 0.1 degrees relative to the cIMU and motion capture measurements, respectively (no movement).

For the sinusoidal profile (Figure 4, Table 3), our developed system showed good tracking capabilities at movement velocities less than 180 degrees per second as reflected in the low RMSE value (RMSE < 2 degrees and RMSE < 8 degrees relative to cIMU and motion capture, respectively). At a movement velocity of 180 degrees per second, the RMSE values increased to 6 degrees and 14 degrees relative to the cIMU and motion capture measurements, respectively. For the stationary axes, there was no noticeable difference in the angles measured by the WIbS, cIMU, and motion capture systems, i.e., no movement (RMSE < 1 degree).

For both movements, our results show that the WIbS may be used to track single step kinematics with an error of less than one degree, relative to both the cIMU and motion capture systems. Tracking cyclic movements that are reflective of gait resulted in a greater error; furthermore, the results also indicate that the WIbS is better at tracking slower speeds, with optimal performance at around 120 degrees per second.

### 3.2. Case Study: Above-Knee Prosthesis User

The developed system was used to activate the vibration motor through the detection of single-axis movement of the robotic arm. Note that the triggering system was affected by a delay of 50 ms, ensuring reliable wireless communication of the movement information.

**Figure 5:**
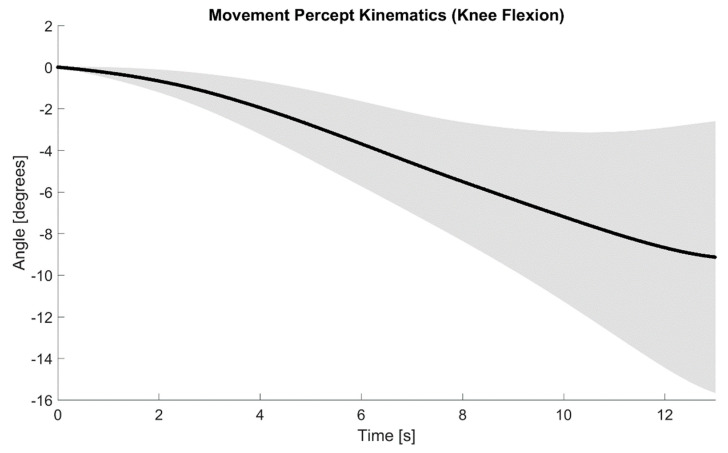
The experienced movement percepts of the participant, as demonstrated by the participant’s intact limb and captured via motion capture. The negative joint angle values (*y*-axis, in degrees) indicate knee flexion. Only 13 s are shown for the *x*-axis since the participant did not experience a change in illusion beyond that time frame. The black line is the average of the 16 successful trials, whereas the shaded area depicts the variation across trials (±1 standard deviation band). Note that the maximum standard deviation, within the terminal phase was approximately 6.5 degrees. The participant’s movement percepts can be generally described as an inverted sigmoidal curve.

The participant experienced the KI in multiple sites on their residual limb. The site with the most consistent illusion was located on the residual quadriceps muscle. This site was reported to elicit a KI strength of 4 to 5 on the Likert scale and, therefore, used for testing the developed feedback system. Since repeated trials of vibratory stimulation intermittently resulted in the sensation of just a stationary phantom limb, only 16 out of the 20 performed trials evoked the movement percept. The average knee flexion angle (black line) and its variability (shaded area: ±1 standard deviation band) across the 16 trials, demonstrated by the intact limb as the experienced movement percept, are shown in Figure 5. As seen in this figure, the movement percept can be loosely described as an inverted sigmoidal curve, with the initial phase indicating a slow ramp up in the movement sensation, the middle phase exhibiting a faster movement sensation, and the final phase indicating the limb coming to a stop.

## 4. Discussion

In the context of prosthetic applications, practical feedback mechanisms must leverage information regarding the state of the prosthetic device during activities of daily living, e.g., ambulation. While existing prosthetic devices can realize a wearer’s movement intentions, information on the actual movement of the prosthesis is not fed back and utilized in a functional or intuitive way. Through a mobile movement tracking and KI triggering system, we have developed a method to close this sensory feedback loop that can be retrofitted to existing prosthesis systems.

### 4.1. Technical Validation

The tracking capabilities of the movement sensors can be interpreted as a consequence of the sensory fusion algorithm used by each IMU within the WIbS. Sensory fusion algorithms generally gather and combine signals from accelerometers, gyroscopes, and magnetometers to generate data regarding device and/or limb orientation [39,40,41]. The majority of sensor fusion techniques estimate unknown variables (e.g., quaternions) through discrete settings at successive times steps, which are dependent on past estimations and current measures [40,41]. Complementary [42,43,44] and Kalman filters [44,45] are the two main approaches utilized in these fusion algorithms. The BNO05 IMU modules within the WIbS most likely rely on either the Complementary or Kalman filter approach in estimating quaternions used for angle computations. Ultimately, we have shown that the two IMUs within the WIbS can reliably track the single-axis movement of a lower-limb prosthetic device for Gaussian and sinusoidal movement profiles. While such movement profiles and associated velocity and acceleration profiles can broadly mimic stepping and walking activities, respectively, they do not consider prosthesis-specific accelerations and associated motion artifacts experienced in real life. Nonetheless, our results for these two movement profiles suggest that the WIbS’ capabilities exceed those for movement onset/termination detection required to successfully administer the KI with a vibration motor—as was shown in our case study. Consequently, the developed WIbS holds promise for greater control of actuation, which might be advantageous as the KI and its delivery are further developed.

### 4.2. System Performance

The feedback system operated with a delay of roughly 800 ms from triggering to actuation. The majority of the delay within the triggering system was caused by the Vibrasens device itself (over 700 ms), while our developed tracking and triggering system had a delay of less than 100 ms. The delay of over 700 ms in the Vibrasens device, likely caused by the internal transients of the vibration motor, was estimated using an iPhone SE’s high-speed camera (Apple Inc., Cupertino, CA, USA) analyzed, frame by frame, through the Vegas Pro software (MAGIX, Berlin, Germany). The delay of less than 100 ms in the tracking and triggering system is most likely attributed to the computation of quaternions, rotation matrices, and Cardan angles, as well as to the wireless communication delay described earlier.

That said, practical use of this feedback system requires a superior vibration actuator with minimal internal transients. Various studies have indicated that delays in feedback can decrease embodiment of the associated prosthetic device, with a maximum delay of 300 ms resulting in minimal distortion of body ownership [46]. Furthermore, incorrect activation of the system resulting in movement percepts during stationary activities such as standing can lead to loss of balance and injury [47]. Alternatively, the inability of the system to activate promptly during gait movements would forfeit the benefits of the sensory feedback system. Therefore, vibratory actuators capable of eliciting movement percepts with shorter transient times must be developed for any practical implementation of this feedback system. Nonetheless, the developed system was able to reliably detect the movement onset of the single-axis robotic arm and trigger the vibration motor appropriately with no false triggers.

### 4.3. Case Study: Above-Knee Prosthesis User

The KI has been effectively demonstrated to improve functional grasp in upper-limb prosthesis applications [14]. However, the implementation of the KI and the characterization of its impact in the lower limb have not been previously explored. This case study demonstrated a simple application of the KI to automatically and repeatedly induce a knee flexion movement illusion. While the WIbS was able to reliably activate the vibration for multiple trials, the movement illusion was not elicited in some trials, and exhibited great variability in others. Since larger muscle bellies may act as large dampers of vibration, it is possible that not the same muscle spindles are recruited every trial [15], potentially affecting the success rate and increasing the variability across trials. To avoid this issue, vibrating a muscle tendon may be a more precise way of promoting a better transmission of the vibration to the muscle spindles, compared to vibrating a muscle belly that is buried under other tissues. However, since individuals with lower limb loss may experience loss or inaccessibility of muscle tendons, the variability in KI remains a notable limitation.

It is worth noting that the participant in this study experienced the KI, in the direction of knee flexion, up to a maximum of only 16 degrees. However, the perception of even a relatively small angular displacement would allow a prosthesis user to obtain critical information on knee kinematics: (1) between heel strike and contralateral toe-off; and (2) just before the initial swing phase. Having access to such phase-based sensory information has been linked to improvements in gait measures in novice users of a transfemoral prosthetic leg that correlate with fall risk—particularly stride length variability, step width variability, and trunk sway variability [48]. At the same time, we were able to modulate, in a recent study [9], the magnitude of the KI by combining vibration of a muscle belly with a skin stretch in a person with transtibial limb loss. In that study, stretching the skin while vibrating a muscle belly on the residual limb resulted in an increase in range and speed of the illusory movement triggered by muscle vibration. If such results are confirmed in a larger sample size, adopting the skin stretch in individuals with transfemoral limb loss may have similar benefits.

Another important consideration is that the KI is a psychophysical phenomenon; hence, other factors will come into play when vision and reciprocal muscle activation are introduced [8,13]. Nonetheless, the presented work in one participant is an essential first step in demonstrating that prosthesis joint movement can be used to actuate the vibration-induced KI, stimulating further investigation of the KI using this technology.

### 4.4. Limitations and Future Developments

**Limitations:** One limitation of this work is that sensor drift of both the proximal and distal movement sensors may lead to inaccurate detection of movement, resulting in false triggers. In practical applications, false movement detection due to drift might be prevented by exploiting contact of the prosthetic foot with the ground. This solution can be accomplished by recalibrating the gyroscope of the distal WIbS when the prosthetic foot is in contact with the ground (i.e., velocity nearly zero), which can greatly reduce drift [49,50]. However, the reduction of gyroscopic drift within IMUs remains an active field of research. Another limitation relates to the Vibrasens vibratory actuator as it is fairly large and was found to be responsible for the majority of the delay in the feedback system (over 700 ms of the roughly 800 ms total). While the Vibrasens actuator has been a common choice for KI experiments in the past (e.g. [9]), future work should evaluate other existing options (e.g., voice coil system VCS1010, Equip-Solutions, Sunnyvale, CA, USA [7]) and also invest into novel designs. Furthermore, due to the stage of this work, i.e., bench testing and case demonstration, we did not consider motion artifacts experienced in real life. The inclusion of such influences will, however, be critical for the next stage of system evaluation. Finally, we should note that, in this work, the KI was investigated for the knee joint, which may not be the best for providing optimal information to the user for balance. Motivated by our recent case study [9], future work will investigate the possibility of consistently eliciting the KI for the ankle joint in individuals with transtibial limb loss.

**Future developments:** The practical utility of the developed sensory feedback system requires implementation and testing with a fully functional prosthesis. Adopting techniques used to integrate customized vibration devices in individuals with upper limb loss [14] may allow for the development of a feedback system for individuals with lower limb loss that also reduces the time delay in the system. However, a variety of practical challenges remain before socket integration of this feedback system is viable. For example, the size of the vibratory actuator(s) presents challenges for socket integration. Furthermore, locations on the residual limb where both the feedback device and prosthetic socket require strategic contact will present a unique challenge for socket design. Additionally, a practical implementation of the KI has to mitigate the fact that the nervous system becomes desensitized with continuous and prolonged exposure to a stimulus, e.g., by only providing intermittent vibration that is functionally relevant. Finally, a functional protocol for quantifying the impact of kinesthetic feedback in lower-limb prostheses, a key component of evaluation, needs yet to be developed and validated.

## Figures and Tables

**Table 1:** To test the performance of the single-axis joint angle tracking system, two movement profiles, Gaussian and sinusoidal, were chosen. For each profile, three different velocities were tested.

Movement Profile	Sets	Type	Amplitude	Frequency	Deviation (σ)
Gaussian	3	Displacement	+90 degrees	N/A	0.083 (s) 3(σ) = 0.25 (s)
		Maximum Velocity	60/120/180 degrees/s		
Sinusoid	3	Displacement	±90 degrees	0.67/1.33/2.00 (Hz)	N/A
		Maximum Velocity	60/120/180 degrees/s		

**Table 2:** Accuracy and repeatability results for the Gaussian movement profile. Shown is the root-mean-square error (RMSE) for the three different movement axes and three different movement velocities: 60 degrees/s, 120 degrees/s, and 180 degrees/s.

Maximum Velocity	Moving Axis	RMSE (Degrees)		Stationary Axis	RMSE (Degrees)	
		Commercial IMU	Motion Capture		Commercial IMU	Motion Capture
Slow (60 degrees/s)	*X*-axis	0.66 ± 0.05	0.26 ± 0.02	*Y*-axis	0.03 ± 0.00	0.03 ± 0.00
				*Z*-axis	0.23 ± 0.00	0.01 ± 0.00
	*Y*-axis	0.64 ± 0.07	0.26 ± 0.02	*X*-axis	0.23 ± 0.00	0.01 ± 0.00
				*Z*-axis	0.03 ± 0.00	0.03 ± 0.00
	*Z*-axis	0.67 ± 0.06	0.26 ± 0.02	*X*-axis	0.24 ± 0.00	0.01 ± 0.00
				*Y*-axis	0.03 ± 0.00	0.03 ± 0.00
Medium (120 degrees/s)	*X*-axis	0.63 ± 0.05	0.15 ± 0.02	*Y*-axis	0.03 ± 0.00	0.03 ± 0.00
				*Z*-axis	0.05 ± 0.00	0.01 ± 0.00
	*Y*-axis	0.61 ± 0.05	0.15 ± 0.02	*X*-axis	0.05 ± 0.00	0.01 ± 0.00
				*Z*-axis	0.03 ± 0.00	0.03 ± 0.00
	*Z*-axis	0.66 ± 0.05	0.15 ± 0.01	*X*-axis	0.05 ± 0.00	0.01 ± 0.00
				*Y*-axis	0.03 ± 0.00	0.03 ± 0.00
Fast (180 degrees/s)	*X*-axis	0.87 ± 0.07	0.28 ± 0.02	*Y*-axis	0.02 ± 0.00	0.02 ± 0.00
				*Z*-axis	0.25 ± 0.00	0.03 ± 0.00
	*Y*-axis	0.88 ± 0.08	0.28 ± 0.02	*X*-axis	0.26 ± 0.00	0.03 ± 0.00
				*Z*-axis	0.02 ± 0.00	0.02 ± 0.00
	*Z*-axis	0.88 ± 0.07	0.28 ± 0.02	*X*-axis	0.25 ± 0.00	0.03 ± 0.00
				*Y*-axis	0.02 ± 0.00	0.02 ± 0.00

**Table 3:** Accuracy and repeatability results for the sinusoidal movement profile. Shown is the root-mean-square error (RMSE) for the three different movement axes and three different movement velocities: 60 degrees/s, 120 degrees/s, and 180 degrees/s.

Maximum Velocity	Moving Axis	RMSE (Degrees)		Stationary Axis	RMSE (Degrees)	
		Commercial IMU	Motion Capture		Commercial IMU	Motion Capture
Slow (60 degrees/s)	*X*-axis	1.54 ± 0.07	5.71 ± 0.08	*Y*-axis	0.03 ± 0.00	0.43 ± 0.00
				*Z*-axis	0.68 ± 0.00	0.28 ± 0.00
	*Y*-axis	1.49 ± 0.05	7.98 ± 0.09	*X*-axis	0.69 ± 0.00	0.29 ± 0.00
				*Z*-axis	0.03 ± 0.00	0.43 ± 0.00
	*Z*-axis	1.58 ± 0.06	4.24 ± 0.04	*X*-axis	0.68 ± 0.00	0.28 ± 0.00
				*Y*-axis	0.03 ± 0.00	0.43 ± 0.00
Medium (120 degrees/s)	*X*-axis	1.29 ± 0.03	5.24 ± 0.04	*Y*-axis	0.20 ± 0.00	0.65 ± 0.00
				*Z*-axis	0.46 ± 0.00	0.17 ± 0.00
	*Y*-axis	1.26 ± 0.04	7.62 ± 0.07	*X*-axis	0.46 ± 0.00	0.17 ± 0.00
				*Z*-axis	0.21 ± 0.00	0.67 ± 0.00
	*Z*-axis	1.29 ± 0.04	3.58 ± 0.06	*X*-axis	0.49 ± 0.00	0.17 ± 0.00
				*Y*-axis	0.19 ± 0.00	0.64 ± 0.00
Fast (180 degrees/s)	*X*-axis	5.32 ± 0.04	11.48 ± 0.06	*Y*-axis	0.14 ± 0.00	0.54 ± 0.00
				*Z*-axis	0.40 ± 0.00	0.19 ± 0.00
	*Y*-axis	5.36 ± 0.49	13.48 ± 0.05	*X*-axis	0.38 ± 0.00	0.19 ± 0.00
				*Z*-axis	0.14 ± 0.00	0.54 ± 0.00
	*Z*-axis	5.58 ± 0.07	9.32 ± 0.03	*X*-axis	0.42 ± 0.00	0.19 ± 0.00
				*Y*-axis	0.14 ± 0.00	0.54 ± 0.00

## Data Availability

The technical data presented in this study are available in the provided figures and tables. The data on the case study are available on request from the corresponding author. These data are not publicly available due to privacy restrictions.
